# Preventing Child Sexual Abuse in the ‘Dunkelfeld’: A Public Health Imperative Requiring Context-Appropriate Science—A Response to König (2025)

**DOI:** 10.1007/s10935-025-00859-6

**Published:** 2025-06-27

**Authors:** K. M. Beier, M. von Heyden, J. Nentzl, T. Amelung

**Affiliations:** Institute of Sexology and Sexual Medicine, Charité—Universitätsmedizin Berlin, Berlin, Germany

## Abstract

König's (2025) critique of the German Prevention Project Dunkelfeld mis-applies standards developed for adjudicated ("Hellfeld") offenders to a voluntary, non-forensic prevention setting. We clarify why context-appropriate science is essential for evaluating interventions that reach individuals whose child sexual abuse (CSA) behaviour remains hidden from the justice system. First, the large gap between official recidivism and high self-reported offending is not methodological failure but strong evidence of the well-documented "dark figure" of undetected sexual crime. Second, Germany's unique legal framework (§ 65d SGB V; § 203 StGB) enables confidential, face-to-face treatment and pharmacological options that are infeasible in jurisdictions with mandatory reporting, making Dunkelfeld programmes a public-health imperative. Third, forensic risk tools and the CODC (2007) guidelines lack demonstrated validity for voluntary help-seekers; carefully collected self-report and dynamic risk-factor change therefore constitute the only context-relevant outcome metrics. Fourth, concerns about iatrogenic effects must be interpreted through the Risk-Need-Responsivity lens: specialised, multi-modal interventions such as the Berlin Dissexuality Therapy (BEDIT) show beneficial changes in cognitive and behavioural risk markers, mirroring findings from recent RCTs of internet-delivered CBT and pharmacological therapy. Finally, all German model sites are currently undergoing an independent, multi-year evaluation commissioned by the national health-insurance association. We conclude that preventing CSA and CSAM requires embracing rigorous yet context-sensitive methodologies rather than importing standards that overlook the dark figure. The ethical imperative to prevent harm and offer help to those at risk persists even acknowledging that no intervention guarantees universal success, demanding our most contextually sensitive scientific efforts.

We appreciate the scientific engagement initiated by König's (2025) critique of the follow-up article on the German Prevention Project Dunkelfeld (Beier et al., 2024). Such critical discourse is indispensable for advancing prevention science. However, it can be asserted that the critique fundamentally misapplies methodological standards and expectations derived from the study of adjudicated offenders (the ‘Hellfeld’—the realm known to the justice system) to the distinct context of a voluntary, non-forensic, preventive 'Dunkelfeld' setting (the 'dark field' of undetected behaviour and individuals outside judicial processes). This oversight neglects the specific public health rationale, the unique legal advantages inherent in the German approach, and the necessary, context-appropriate methodological considerations required for evaluating interventions designed to prevent Child Sexual Abuse (CSA) and the use of Child Sexual Abuse Material (CSAM) among those not yet formally detected. Underpinning these efforts is the profound societal and ethical imperative to protect potential victims and offer pathways to change, even when faced with complex behaviors and the inherent challenges of intervention in a hidden population—an imperative that drives both 'Dunkelfeld' prevention and the broader societal approaches to 'Hellfeld' treatment.

## The Discrepancy Between Official Data and Self-Report: Overwhelming Empirical Evidence of the Dark Figure

König frames the stark divergence between low official criminal recidivism rates (0% for CSA in the specific N = 56 follow-up cohort; Beier et al., 2024) and high self-reported delinquency (7.7% CSA recidivism; 89.1% CSAM recidivism) as a critical flaw, suggesting methodological failure and the unreliability of self-report. It can be strongly contended that this discrepancy is not a methodological failure but robust, consistent empirical evidence reflecting the vast, well-documented 'dark figure'—the substantial amount of sexual offending missed by official criminal justice statistics.

Official records capture only the 'tip of the iceberg'. Research using comprehensive German registry data (Hörnle et al., 2024) confirmed minimal official recidivism (1.1% for CSA, 5.7% for CSAM after 6 years). This low detection rate starkly contrasts with self-reported data from relevant populations.

Studies accessing the Dunkelfeld consistently reveal much higher rates of actual behaviour. Von Franqué et al. (2023), in a comparable German Dunkelfeld sample (N = 165), found a self-reported CSAM recidivism rate of 39%—11 times higher than the expected official rate—and a significant self-reported CSA rate of 14%, comparable to official rates for convicted contact offenders. The follow-up (Beier et al., 2024; based on Nentzl & Scherner, 2021) showed 89.1% CSAM recidivism. Crucially, Schuler et al. (2021), analyzing data from over 4100 users of the anonymous “Troubled Desire” web app, found that among users reporting sexual interest in children, 41.5% reported lifetime CSA offenses and 72.8% reported lifetime CSAM use, with the explicit finding: “the majority of the offenses were not known to legal authorities.” Lahtinen et al. (2025) large-scale study further confirmed significant ongoing high-risk behaviors even among previously charged individuals seeking CSAM anonymously online. Studies like Beier (1998) also documented high rates (50–80%) of undetected reoffending decades ago in forensic-adjacent samples.

This undeniable gap between official detection and actual behaviour validates Dunkelfeld prevention strategies. Self-reported data, while imperfect and requiring careful interpretation due to inherent limitations (e.g., recall, social desirability; Podsakoff et al., 2003), offers an indispensable window into the hidden reality these programs address, a reality otherwise inaccessible. Nevertheless, in the specific context of voluntary prevention programs where official records are demonstrably insufficient for capturing the phenomena of interest, methodologically sound collection and cautious analysis of self-report remain essential for evaluating intervention impact.

## The Public Health Imperative and the Unique German Context

The necessity for proactive, preventive strategies is amplified by the alarming global increase in detected CSA and the exponential proliferation of CSAM online (as noted in Beier et al., 2024; König, 2025). A purely reactive judicial system is insufficient. Indicated prevention—offering support and treatment to individuals who recognize their risk related to Pedophilic or Hebephilic Disorder (PHD) and voluntarily seek help—is crucial. Germany’s approach, operationalized through the “Kein Täter werden” network and similar initiatives, represents a significant public health effort enabled by a unique legal framework: § 65d SGB V (Social Code V) mandates the funding of model projects for the prevention and treatment of pedophilic sexual disorders, ensuring structural support. § 203 StGB (German Criminal Code) provides strong professional confidentiality protection for therapists.

As König notes, this confidentiality is central. However, rather than being a methodological weakness, it is a fundamental strength enabling the German Dunkelfeld approach. Unlike jurisdictions with broad mandatory reporting laws that often deter help-seeking or limit interventions primarily to anonymous online formats (Lätth et al., 2022; Schuler et al., 2021), the German framework facilitates intensive, face-to-face therapeutic engagement. This personal contact is paramount for building trust, addressing complex psychological issues (including comorbidities and dynamic risk factors like offense-supportive attitudes), assessing real-world risk situations, facilitating the voluntary use of androgen deprivation therapy with informed consent when indicated, and implementing proactive, integrated child protection measures directly within the therapy, as detailed in specific modules of the Berlin Dissexuality Therapy (BEDIT) manual (e.g. Grundmann et al., 2021). This potential for deeper, personalized, and safer intervention represents a significant advantage compared to approaches constrained by different legal environments.

## Methodological Appropriateness: Context Dictates Methods

König's critique hinges on applying Hellfeld standards and tools to the Dunkelfeld. This is methodologically inappropriate. Voluntary help-seekers differ from adjudicated offenders (Lahtinen et al., 2025; von Franqué et al., 2023). Standard forensic risk assessment tools, validated for predicting official recidivism, show poor predictive validity for self-reported behavior in this group (von Franqué et al., 2023).

The international Delphi study by Stephens et al. (2024) revealed significant expert contention regarding the direct transfer of specific forensic assessment and risk-reduction techniques to non-mandated settings, while finding high consensus for general clinical practices.

König invokes the Collaborative Outcome Data Committee (CODC, 2007) guidelines as the “gold standard.” However, these were explicitly designed for evaluating treatment for adjudicated sexual offenders, using official recidivism. Applying this standard rigidly to preventive work with self-reported outcomes misinterprets the guidelines' scope. While CODC appropriately assigns lower confidence ratings to 'self-reported only' recidivism information (p. 69), the guidelines themselves acknowledge that 'the choice of outcome variable has consequences for rating study quality' (p. 1). In the Dunkelfeld, where official records systematically fail to capture the population of interest, carefully collected self-report is not merely an alternative but a necessary, contextually relevant measure—the only methodology capable of providing insight into the 'dark figure' of undetected behavior. The Lätth et al. (2022) Randomized Controlled Trial (RCT) successfully used self-reported CSAM viewing time as its primary outcome, demonstrating its acceptance in rigorous, context-appropriate research. The need for context-specific evaluation is increasingly recognized, with major international bodies like the European Union now developing bespoke evaluation toolkits for Child Sexual Abuse and Exploitation prevention programs, distinct from traditional forensic measures (Di Gioia et al., 2025).

The Gottfredson et al. (2015) standards, while foundational, advocate for rigor and methodological evolution appropriate to the stage of prevention science research.

## Contextualizing Iatrogenic Effects and Measuring Multi-Modal Effectiveness

König raises concerns about iatrogenic effects, citing Holper et al. (2024). This requires careful contextualization within the Risk-Need-Responsivity (RNR) framework, which Holper et al.'s findings support:

- Treatment benefit is moderated by risk level (positive for medium/high-risk based on official reconviction). Dunkelfeld participants exhibit high self-reported risk, making simple "low-risk" categorization based on actuarial tools potentially misleading (von Franqué et al., 2023).
- Specialized treatment programs were significantly more effective than non-specialized ones. Extrapolating potential harm from non-specialized settings is inappropriate.

Contrary to blanket skepticism, evidence suggests interventions can be effective. The Lätth et al. (2022) RCT showed a significant reduction in self-reported CSAM viewing via internet-based CBT in an anonymous online sample. The Landgren et al. (2020) RCT demonstrated that pharmacological intervention (Degarelix) significantly reduced dynamic risk factor scores in help-seeking men with PHD. The study by Beier et al. (2024) showed significant improvements in specific dynamic cognitive factors (cognitive victim empathy, CSAM-supportive attitudes) during treatment, with the latter persisting at follow-up. Measuring such dynamic factors provides key, proximal indicators of treatment impact relevant to prevention, supplementing the distal and often inaccessible measure of official recidivism.

The critique overlooks the comprehensive nature of the therapy evaluated. The BEDIT manual (Beier 2021) explicitly includes pharmacological treatment options as an adjunct to psychotherapy, particularly for individuals struggling with impulse control or high sexual preoccupation. This multi-modal approach allows for tailored interventions based on individual need and risk.

The persistent underlying risk of PHD (Beier, 1998) remains a significant factor, but evidence indicates that comprehensive, specialized interventions can positively impact relevant outcomes.

## Anonymity, Access, and Rigorous Scientific Evaluation

König uses the Zurich study (de Tribolet-Hardy et al., 2024) to question anonymity. The Swiss BSV (2025) report clarifies this occurred in a unique, non-representative "Best Practice Modell." Anonymity remains key for initial helpline access elsewhere in Switzerland and is a cornerstone of the German Dunkelfeld approach, legally supported by § 65d SGB V and § 203 StGB. Critically, the German model projects operating under this mandate are subject to a comprehensive, independent, long-term external scientific evaluation (2018–2026) by the Professur für Klinische Psychologie und Psychotherapie, TU Chemnitz (n.d.), commissioned by the GKV-Spitzenverband. This evaluation employs a multi-phase, mixed-methods design, including process and outcome assessments utilizing quantitative and qualitative data collected from program participants, therapists, and project documentation across all participating model projects (Mühlig, 2018). Its scope covers the analysis of therapeutic processes, participant characteristics, treatment adherence, effectiveness regarding prevention goals, organizational structures, and cost-effectiveness. Notably, this evaluation framework incorporates a large-scale, multi-center RCT specifically investigating the effectiveness of BEDIT among voluntary help-seekers (Beier, 2024). This embedded RCT focuses on primary outcomes such as changes in dynamic risk factors (e.g., offense-supportive attitudes, victim empathy, coping skills) and self-reported offending behaviors. The overall evaluation, including the RCT, aims to generate robust, evidence-based recommendations for the optimization and potential nationwide implementation of these preventive services under § 65d SGB V (Mühlig, 2018).

## Conclusion: A Public Health Imperative Requires Context-Appropriate Science

Preventing CSA and CSAM demands a multi-faceted public health strategy that includes indicated prevention through Dunkelfeld programs. Evaluating these initiatives requires methodologies sensitive to their unique context. The well-documented discrepancy between official and self-reported data (as detailed in Section “The Discrepancy Between Official Data and Self-Report: Overwhelming Empirical Evidence of the Dark Figure”) robustly validates the need for these programs by revealing the true extent of the 'dark figure.' As argued (Section “Methodological Appropriateness: Context Dictates Methods”), standard forensic tools and evaluation criteria are ill-suited for Dunkelfeld settings, lacking consensus and demonstrated validity for these unique populations and their preventive objectives. Consequently, evaluating Dunkelfeld programs necessitates the careful use of self-report and alternative outcome measures, such as changes in dynamic risk factors, for which initial evidence indicates positive intervention effects (see Section “Contextualizing Iatrogenic Effects and Measuring Multi-Modal Effectiveness”). Concerns about iatrogenic effects, as discussed (Section “Contextualizing Iatrogenic Effects and Measuring Multi-Modal Effectiveness”), must be carefully contextualized by participant risk level and treatment specialization. Germany's distinctive legal framework underpins a comprehensive approach, including intensive in-person therapy and pharmacotherapy, which is subject to the rigorous, ongoing external scientific evaluation detailed previously (Section “Anonymity, Access, and Rigorous Scientific Evaluation”).

The ethical imperative is to utilize the best available and appropriate science, informed by emerging expert consensus and grounded in prevention science principles, to understand and improve these important preventive services. Ultimately, the scientific and methodological debates occur within a larger societal consensus: that we have a profound ethical obligation to strive to prevent CSA and offer help to those who pose a risk, whether they are known to the justice system or are part of the 'dark figure' seeking to avoid causing harm. This obligation persists even acknowledging that no intervention can guarantee universal success, demanding our most rigorous and contextually sensitive efforts.

While the public health imperative for Dunkelfeld prevention is clear, the scientific discourse rightly scrutinizes the methodological approaches to evaluating these specialized programs. Our response seeks to demonstrate that the methods employed, particularly the necessary reliance on self-report to access the 'dark figure,' are contextually appropriate and part of an evolving scientific endeavor to understand and improve these vital services, rather than to imply that such scrutiny itself is counterproductive. We thank König (2025) for identifying the error in Figure 1 of our original publication. The corrected breakdown is as follows: of the 126 treatment completers, exclusions included 16 individuals with a follow-up period of less than one year, 39 (not 40) with no valid contact information, 1 deceased, 11 who refused, and 3 who retracted their participation, resulting in the correct total of 56 participants. This error does not affect any of the analyses or conclusions presented.
